# Cisplatin Pharmacodynamics Following Endobronchial Ultrasound-Guided Transbronchial Needle Injection into Lung Tumors

**DOI:** 10.1038/s41598-019-43270-y

**Published:** 2019-05-02

**Authors:** Vitor Mori, Gregory S. Roy, Jason H. T. Bates, C. Matthew Kinsey

**Affiliations:** 1 Department of Medicine, University of Vermont College of Medicine, Burlington, VT 05405 USA; 2 Department of Telecommunications and Control Engineering, University of Sao Paulo, Sao Paulo, Brazil

**Keywords:** Therapeutic endoscopy, Non-small-cell lung cancer

## Abstract

Intratumoral delivery of cisplatin by endobronchial ultrasound-guided transbronchial needle injection (EBUS-TBNI) has recently emerged as a therapy for treating peribronchial lung cancers. It remains unclear, however, where best to inject drug into a tumor, and at how many sites, so current cisplatin delivery strategies remain empirical. Motivated by the need to put EBUS-TBNI treatment of lung cancer on a more objective footing, we developed a computational model of cisplatin pharmacodynamics following EBUS-TBNI. The model accounts for diffusion of cisplatin within and between the intracellular and extracellular spaces of a tumor, as well as clearance of cisplatin from the tumor via the vasculature and clearance from the body via the kidneys. We matched the tumor model geometry to that determined from a thoracic CT scan of a patient with lung cancer. The model was calibrated by fitting its predictions of cisplatin blood concentration versus time to measurements made up to 2 hrs following EBUS-TBNI of cisplatin into the patient’s lung tumor. This gave a value for the systemic volume of distribution for cisplatin of 12.2 L and a rate constant of clearance from the tumor into the systemic compartment of 1.46 × 10^−4^ s^−1^. Our model indicates that the minimal dose required to kill all cancerous cells in a lung tumor can be reduced by roughly 3 orders of magnitude if the cisplatin is apportioned between 5 optimally spaced locations throughout the tumor rather than given as a single bolus to the tumor center. Our findings suggest that optimizing the number and location of EBUS-TBNI sites has a dramatic effect on the dose of cisplatin required for efficacious treatment of lung cancer.

## Introduction

Cisplatin is widely used to treat lung cancer because of its ability to interfere with cell replication^1–3^. The usual route of administration is intravenous (IV). This exposes normal cells throughout the body to the toxicity of the drug, resulting in a heavy side-effect burden in off-target tissues. Moreover, cisplatin is taken up more readily by normal cells^4^ compared to tumor. For this reason, we and others have recently begun to explore the therapeutic potential of directly injecting cisplatin into lung tumors that are adjacent to airways accessible by bronchoscopy using an approach known as endobronchial ultrasound-guided transbronchial needle injection (EBUS-TBNI)^5,6^. This presumably allows higher drug concentrations to be achieved within the tumor while at the same time reducing both systemic concentrations and side effects. However, we currently have little data to guide the choice of injection site(s) within the tumor, or the dose per site. This leaves treatment planning for direct cisplatin injection into lung tumors on an entirely empirical footing, along with its attendant risks and missed opportunities.

Accordingly, we hypothesized that an anatomically-based mathematical/computational model of cisplatin dynamics within a peribronchial lung tumor could be useful in the design of therapeutic injection strategies, and could specifically address the question of the optimal number of injections to be performed. Here we develop such a model and apply it to a high-resolution computed tomography (CT) scan of a patient’s lung tumor. We show that by accounting for intratumoral diffusion of drug between extra-and intracellular compartments, as well as its convective clearance via the vasculature, we can move toward a rationale design for direct lung cancer treatment strategies using EBUS-TBNI of cisplatin.

## Materials and Methods

### Model development

**Figure 1:**
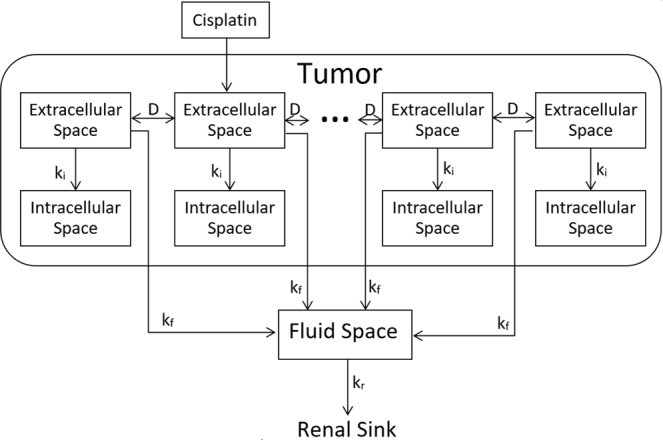
Model structure. Each labeled rectangle represents a single well-mixed compartment.

We assume that a tumor can be represented as a superposition of two distinct spaces, the *extracellular space* and the *intracellular space*, both of which are assumed to have volumes that do not change over the timescale of the model. The extracellular space consists of the interstitial fluid and connective tissue within a tumor, while the intracellular space consists of the cytoplasm and associated organelles, including the nuclei, of the malignant cells. The small blood vessels that perfuse the tumor are contiguous with, and thus part of, a separate *fluid space* that includes the systemic vasculature and possibly also some extravascular spaces of distribution within the tissues of the body. Cisplatin is eventually excreted from the body, predominately via the kidneys. For the purposes of developing a continuous mathematical theory of the model, we assume that the extracellular and intracellular spaces comprise, within each infinitesimal volume of tumor, two topographically coincident but functionally distinct compartments occupying volume fractions of ${\alpha }_{e}$ and ${\alpha }_{i}$, respectively, where ${\alpha }_{e}+{\alpha }_{i}=1$. The model thus consists of a series of interconnected pairs of extracellular and intracellular compartments that link to a single fluid compartment as illustrated in Fig. 1.

When cisplatin is first injected transbronchially into a tumor it enters the extracellular space at the site of injection from which it proceeds to diffuse throughout the rest of the extracellular space with diffusion coefficient $D$. The cisplatin subsequently leaves the extracellular space by flowing into the intracellular and fluid spaces within the tumor with rate-constants per unit volume of ${k}_{i}$ and ${k}_{f}$, respectively. In the intracellular space it binds immediately and irreversibly to the cell DNA, so we assume that the intracellular space acts as a sink for cisplatin. When cisplatin passes into the blood vessels it is rapidly convected into the rest of the fluid space where it mixes with the blood over a time-scale of minutes, which is short compared to the time-scale of the model. We thus consider the fluid space to act as a single well-mixed compartment. Cisplatin diffuses from the fluid space back into the extracellular space with rate constant $k^{\prime}$_*f*_.

Based on the above considerations, the concentration of cisplatin in the extracellular space, ${\phi }_{e}(\overrightarrow{r},t)$, is governed by the equation

1 $$\frac{d{\phi }_{e}(\overrightarrow{r},t)}{dt}=D{\nabla }^{2}{\phi }_{e}(\overrightarrow{r},t)-({k}_{i}+{k}_{f}){\phi }_{e}(\overrightarrow{r},t)+{k^{\prime} }_{f}{\phi }_{f}(t)$$

where ${\phi }_{f}(t)$ is the concentration of cisplatin in the fluid space. The fluid space constitutes a single well-mixed compartment so ${\phi }_{f}(t)$ is a function purely of time, and is governed by

2 $$\frac{d{\phi }_{f}(t)}{dt}=\frac{1}{{V}_{f}}\frac{d[{\int }_{V}{k}_{f}{\phi }_{e}(\overrightarrow{r},t)d\overrightarrow{r}]}{dt}-{k^{\prime} }_{f}{\phi }_{f}(t)-{k}_{r}{\phi }_{f}(t)$$

where ${k}_{r}$ is the rate-constant for renal excretion and ${V}_{f}$ is the total fluid volume. Finally, cisplatin accumulates locally in the intracellular space from its adjacent extracellular supply, so the intracellular concentration, ${\phi }_{i}(\overrightarrow{r},t)$, is governed simply by

3 $$\frac{d{\phi }_{i}(\overrightarrow{r},t)}{dt}={k}_{i}{\phi }_{e}(\overrightarrow{r},t).$$

When cisplatin is injected directly into a tumor the above equations can be simplified by noting that ${\phi }_{f}(t)$ is always much lower than the early values of ${\phi }_{e}(\overrightarrow{r},t)$, which means that these early values of ${\phi }_{e}(\overrightarrow{r},t)$ are chiefly responsible for generating the clinically effective concentrations of cisplatin in the intracellular space. This allows us to approximate the fluid space as a sink for cisplatin, which reduces Eq. 1 to

4 $$\frac{d{\phi }_{e}(\overrightarrow{r},t)}{dt}=D{\nabla }^{2}{\phi }_{e}(\overrightarrow{r},t)-({k}_{i}+{k}_{f}){\phi }_{e}(\overrightarrow{r},t).$$

Let a dose $M$ of cisplatin be injected at a location in the tumor centered on point ${\overrightarrow{r}}_{i}$ within the extracellular space at $t=0$ such that we can approximate ${\phi }_{e}({\overrightarrow{r}}_{i},0)$ as $M\delta ({\overrightarrow{r}}_{i})$, where $\delta$ is the Dirac delta-function. The solution to Eq. 4, assuming the tumor boundary to be at infinity, is then

5 $${\phi }_{e}(\overrightarrow{r},t)=\frac{M{e}^{-({k}_{i}+{k}_{f})t}}{\sqrt{4\pi {(Dt)}^{3}}}{e}^{\frac{-{|\overrightarrow{r}-{\overrightarrow{r}}_{i}|}^{2}}{4Dt}}.$$

If $M$ is distributed between $N$ different injection locations within the tumor, the superposition principle gives

6 $${\phi }_{e}(\overrightarrow{r},t)=\sum _{j=1}^{N}\frac{{m}_{j}{e}^{-({k}_{i}+{k}_{f})t}}{\sqrt{4\pi {(Dt)}^{3}}}{e}^{\frac{-{|\overrightarrow{r}-{\overrightarrow{r}}_{j}|}^{2}}{4Dt}}$$

where $$M=\sum _{j=1}^{N}{m}_{j}$$.

From Eqs 3 and 6 we then have

7 $$\begin{array}{rcl}{\phi }_{i}(\overrightarrow{r},t) & = & {k}_{i}{\int }_{0}^{t}{\phi }_{e}(\overrightarrow{r},\tau )d\tau \\  & = & {k}_{i}\sum _{j=1}^{N}\frac{{m}_{j}}{2D|\overrightarrow{r}-{\overrightarrow{r}}_{j}|}[{e}^{-2\alpha \beta }(1-{\rm{erf}}(\alpha -\beta ))\\  &  & -\,{e}^{2\alpha \beta }(1-{\rm{erf}}(\alpha +\beta ))]\end{array}$$

where

8 $$\alpha =\sqrt{\frac{{|\overrightarrow{r}-{\overrightarrow{r}}_{j}|}^{2}}{4D\tau }}\,{\rm{and}}\,\beta =\sqrt{({k}_{i}+{k}_{f})\tau }.$$

and $\mathrm{erf}(\alpha +\beta )$ is the error function.

The ability of cisplatin to eradicate cancer depends in some way on its intracellular concentration profile, but exactly how remains a matter of debate. In the interests of avoiding unnecessary complexity we will be guided by the fact that in the cytoplasm the *cis* chlorine groups in the cisplatin molecule are replaced by water molecules^7^, allowing it to bind essentially irreversibly to DNA. This interferes with the ability of DNA both to replicate and to repair itself, eventually leading to cell death by apoptosis^2,3,8^. Cytotoxicity is thus clearly related to the mass accumulation of cisplatin within the nucleus, which under the assumptions described above can be approximated by its asymptotic intracellular concentration ${\phi }_{i}(\overrightarrow{r},t\,\to \,\infty )$.

### Model fitting

We assume that cisplatin does not leave the tumor at its boundary, since there is very little tissue beyond the boundary for it to move into, so the boundary may affect the shape of ${\phi }_{e}(\overrightarrow{r},t)$. However, if we assume that the boundary essentially reflects back into the tumor any drug that would have otherwise diffused beyond it, the total amount of cisplatin remaining in the extracellular space will remain relatively unaffected, in which case we can estimate this total amount by spatially integrating Eq. 6 to infinity to obtain

9 $${M}_{e}(t)={\int }_{V}{\phi }_{e}(\overrightarrow{r},t)dV=\sum _{j=1}^{n}{m}_{j}{e}^{-({k}_{i}+{k}_{f})t}.$$

Similarly, substituting Eq. 6 into Eq. 3 and spatially integrating the result to infinity gives

10 $${M}_{i}(t)=\sum _{j=1}^{N}\frac{{\alpha }_{i}}{{\alpha }_{e}}\frac{{k}_{i}}{{k}_{i}+{k}_{f}}{m}_{j}(1-{e}^{-({k}_{i}+{k}_{f})t}).$$

The amount of cisplatin that has moved from the extracellular space to the fluid space at any point in time is simply the initially injected amount less the amounts in the extracellular space (Eq. 9) and the intracellular space (Eq. 10). That is, from Eq. 2,

11 $${\int }_{V}{k}_{f}{\phi }_{e}(\overrightarrow{r},t)d\overrightarrow{r}=\sum _{j=1}^{N}(1-\frac{{\alpha }_{i}}{{\alpha }_{e}}\frac{{k}_{i}}{{k}_{i}+{k}_{f}}){m}_{j}(1-{e}^{-({k}_{i}+{k}_{f})t}).$$

Finally, we assume that the intracellular space is much smaller than the extracellular space (i.e., ${\alpha }_{e}\gg {\alpha }_{i}$), so Eq. 11 becomes

12 $${\int }_{V}{k}_{f}{\phi }_{e}(\overrightarrow{r},t)d\overrightarrow{r}=\sum _{j=1}^{n}{m}_{j}(1-{e}^{-({k}_{i}+{k}_{f})t}).$$

Fluid cisplatin concentration, ${\phi }_{f}(t)$, is the result of a balance between the flow of drug from the extracellular to the fluid space and the drug clearance rate due to blood filtration by the kidneys^4^, which will be modeled as a sink with time constant ${k}_{r}$. Combining Eqs 2 and 12 gives:

13 $$\frac{d{\phi }_{f}(t)}{dt}=\frac{1}{{V}_{f}}\frac{\partial }{\partial t}(\sum _{j=1}^{n}{m}_{j}(1-{e}^{-({k}_{i}+{k}_{f})t}))-{k}_{r}{\phi }_{f}(t)$$

so

14 $${\phi }_{f}(t)=\sum _{j=1}^{n}\frac{{k}_{f}+{k}_{i}}{{k}_{f}+{k}_{i}-{k}_{r}}\frac{{m}_{j}}{{V}_{f}}({e}^{-{k}_{r}t}-{e}^{-({k}_{i}+{k}_{f})t}).$$

Cisplatin biological half-life in humans has been reported to be aproximately 30 min, so we assign it the value ${k}_{r}=3.85\cdot {10}^{-4}\,{s}^{-1}$.

Equation 14 was fit to the measured cisplatin blood concentrations obtained from the human subject. The fitting was achieved by optimizing the values of the two free parameters $({k}_{f}+{k}_{i})$ and ${V}_{f}$ using a gradient-based algorithm to minimize the cost function

15 $${J}_{Blood}({k}_{f}+{k}_{i},{k}_{{\rm{f}}})=\sqrt{\sum _{N=1}^{5}\,{[{\varphi }_{bloo{d}_{data}}-{\varphi }_{bloo{d}_{model}}]}^{2}}.$$

### Patient data

We maintain an ongoing protocol, approved by the Institutional Review Board of the University of Vermont, to evaluate tissue and correlative data obtained during clinically indicated EBUS-TBNI of cisplatin. All patients referred for potential EBUS-TBNI of cisplatin are reviewed at the Multidisciplinary Lung Tumor Board of the University of Vermont Medical Center to insure that there are no other more well-established therapeutic options. Patients provided informed consent, and all research procedures were conducted in accordandance with Good Clinical Practice (GCP) as outlined by the Collaborative Institutional Training Initiative (CITI).

In order to understand potential toxicities from EBUS-TBNI of cisplatin we performed serial cisplatin blood level monitoring during and following a singe procedure. This allowed us to determine when blood cisplatin levels peaked in order to guide the timing of blood draws in subsequent cases. Five 8 mg cisplatin injections were delivered into the tumor of a patient with recurrent lung cancer. Ultrasound guidance was used to attempt to distribute the 5 injections evenly throughout the tumor over an interval of 18 min. The concentration of cisplatin was measured in venous blood drawn at 5, 15, 30, 60 and 120 min after the final injection.

The patient also underwent a high-resolution computed tomography (CT) scan of the thorax from which the location, size and 3D shape of the lung tumor was accurately determined. We used MATLAB 2015b (The MathWorks, Natick, MA, USA) to create a geomerically accurate representation of the tumor boundary from the CT image.

### Optimizing injection strategies

We assume that tumor cell death occurs when the intracellular concentration of cisplatin reaches a lethal threshold level of ${\phi }_{t}$. There is little guidance in the literature as to the appropriate value of ${\phi }_{t}$ to use in the model, so for our initial simulations we arbitrarily chose a nominal value of 0.5 mg/mL. This is a relatively conservative estimate since it implies that at least half of the delivered agent (20 of the injected 40 mg distributed throughout a 40 ml tumor) must be absorbed into the cell nucleus to be cytotoxic, which will happen in any cell in which ${\phi }_{t}$ is exceeded by the asymptotic value of ${\phi }_{i}(\overrightarrow{r},t)$ given by Eq. 7. This asympototic value is

16 $${\phi }_{i}(\overrightarrow{r},t\to \infty )=\sum _{j=1}^{N}\,\frac{{k}_{i}{m}_{j}}{D|\overrightarrow{r}-{\overrightarrow{r}}_{j}|}\cdot {e}^{-2\alpha \beta }$$

The value of $D$ for a drug in normal tissues at 37 °C is reported to be a function of the drug molecular weight according to the empirical law^9^

17 $$D=1.778\cdot {10}^{-4}\,{(MW)}^{-0.75}\,c{m}^{2}/s.$$

The molecular weight of cisplatin is 300 Da, giving a value of $D$ of 2.47 × 10^−6^ cm^2^ s^−1^.

For EBUS-TBNI to successfully treat a lung tumor, ${\phi }_{i}(\overrightarrow{r},t\,\to \,\infty )$ must exceed ${\phi }_{t}$ everywhere within the tumor. Achieving this condition depends on both the total cisplatin dose, $M$, and the manner in which this dose is apportioned between different injection sites within the tumor. At the same time, it is clearly to the patient’s benefit to have $M$ be as small as possible so that systemic side effects are minimized. Accordingly, based on the model of cisplatin dynamics developed above, we determined the locations and doses of $N$ injections that would minimize $M$ subject to the condition ${\phi }_{i}(\overrightarrow{r},t\,\to \,\infty ) > {\phi }_{t}$ at every point within the tumor, for $N=1,\ldots ,6$. We identified these optimum injection strategies by first using a genetic algorithm to determine the spatial locations of $N$ injections that maximized the minimum value of ${\phi }_{i}(\overrightarrow{r},t\,\to \,\infty )$ within the tumor for specified values of $N$ and $M$. Since the model predictions scale linearly with $M$, finding the minimum value of $M$ was then simply a matter of scaling the doses so that the minimum value of ${\phi }_{i}(\overrightarrow{r},t\,\to \,\infty )$ equaled ${\phi }_{t}$.

## Results

**Figure 2:**
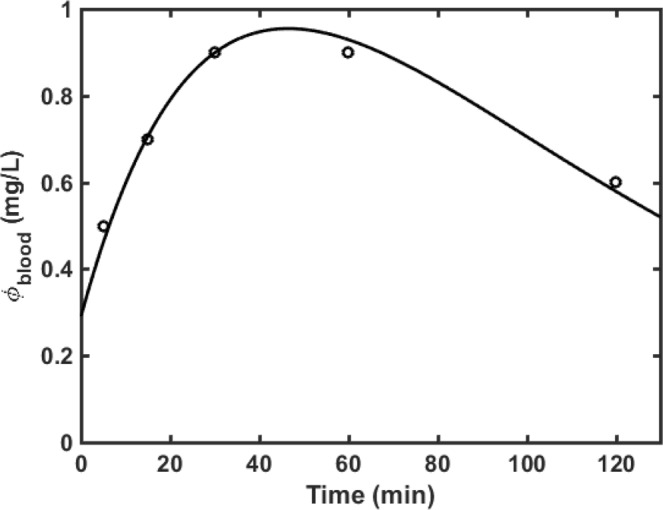
Measured blood concentration of cisplatin (open circles) following five 8-mg intratumoral injections into the lung tumor of a patient. Also shown (solid line) is the fit of Eq. 14 to the data (r^2^ = 0.98).

The fit of Eq. 14 to the data of cisplatin blood concentration versus time after injection is shown in Fig. 2, and yields values for the two independent parameters of ${V}_{f}=12.2$ L and ${k}_{i}+{k}_{{\rm{f}}}=2.51\times {10}^{-4}$ s^−1^. Previous studies in head, neck and gastric carcinomas^10,11^ have reported ${k}_{i}=1.05\times {10}^{-4}$ s^−1^, so if we assume the same to be true for lung carcinomas then we obtain ${k}_{f}=1.46\times {10}^{-4}\,\,$ s^−1^.

**Figure 3:**
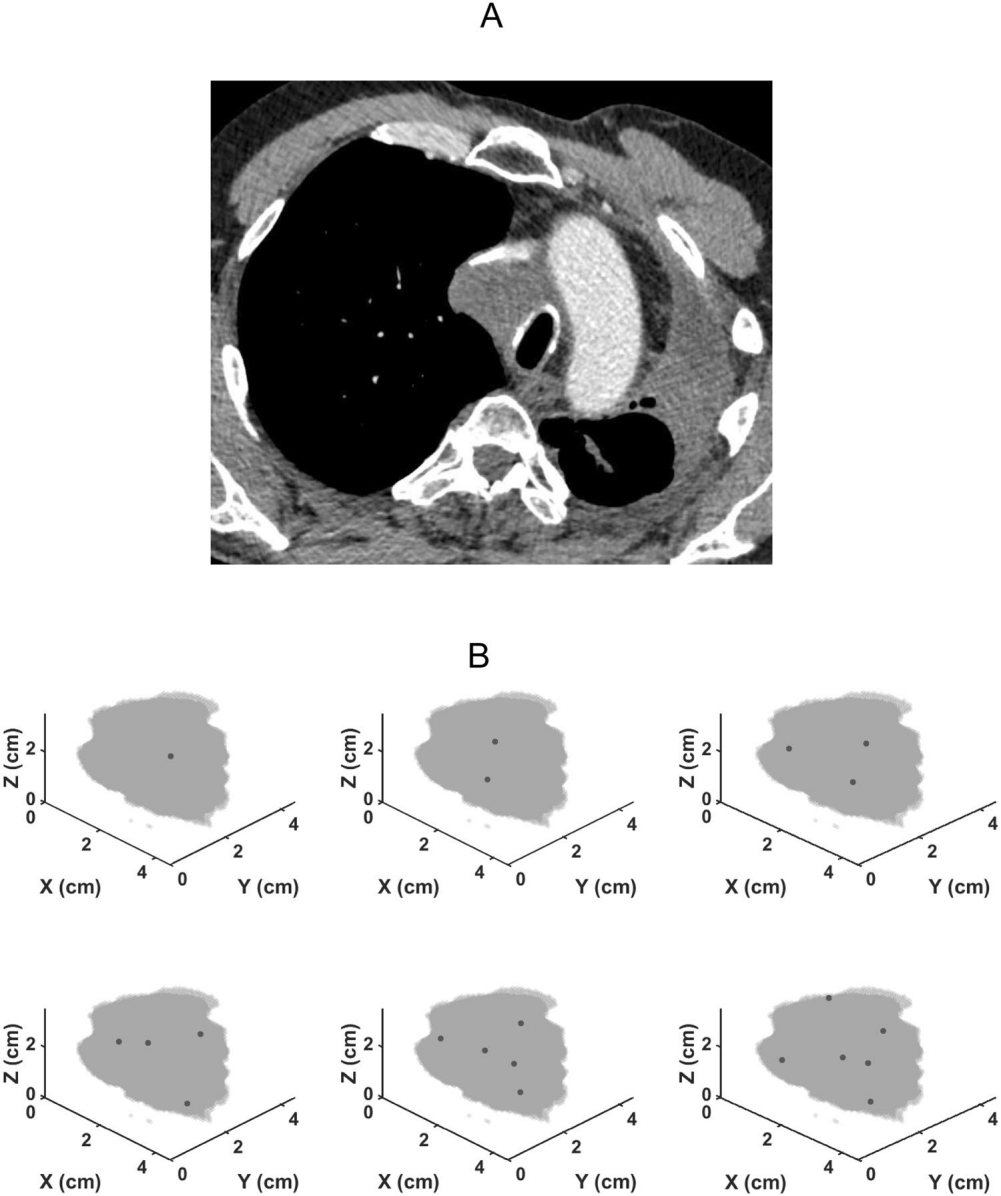
(**A**) Axial CT image demonstrating a right paratracheal lung cancer, occurring in the prior radiation field. (**B**)Three-dimensional reconstructions of the tumor showing the optimal locations for 1 to 6 injections of cisplatin.

**Figure 4:**
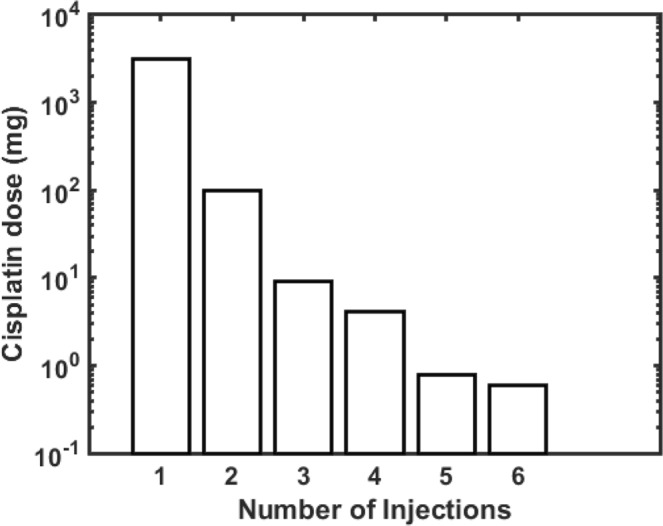
Minimum total cisplatin dose needed for all tumor cells to reach a threshold intracellular concentration of 0.5 × 10^−7^ mg/mL as a function of the number of injections. Equal doses are given with each injection, and the injection sites are located optimally.

Figure 3 shows renditions of the lung tumor in the patient we studied along with the optimal locations of cisplatin injection sites for 1 to 6 injections calculated using the computational model with the values of ${V}_{f}$, ${k}_{i}$ and ${k}_{{\rm{f}}}$ given above. Note that a single injection is optimally located close to the middle of the tumor while multiple injections are distributed in a balanced way thoughout the tumor mass, as one would expect intuitively. The major benefit of multiple injections, however, is evident in Fig. 4 which shows the total cisplatin dose required to kill all tumor cells as a function of the number of injections. This dose decreases by more than 3 orders of magnitude in going from 1 to 5 injections. Relatively little additional dose reduction is achieved by going to 6 injections, however.

**Figure 5:**
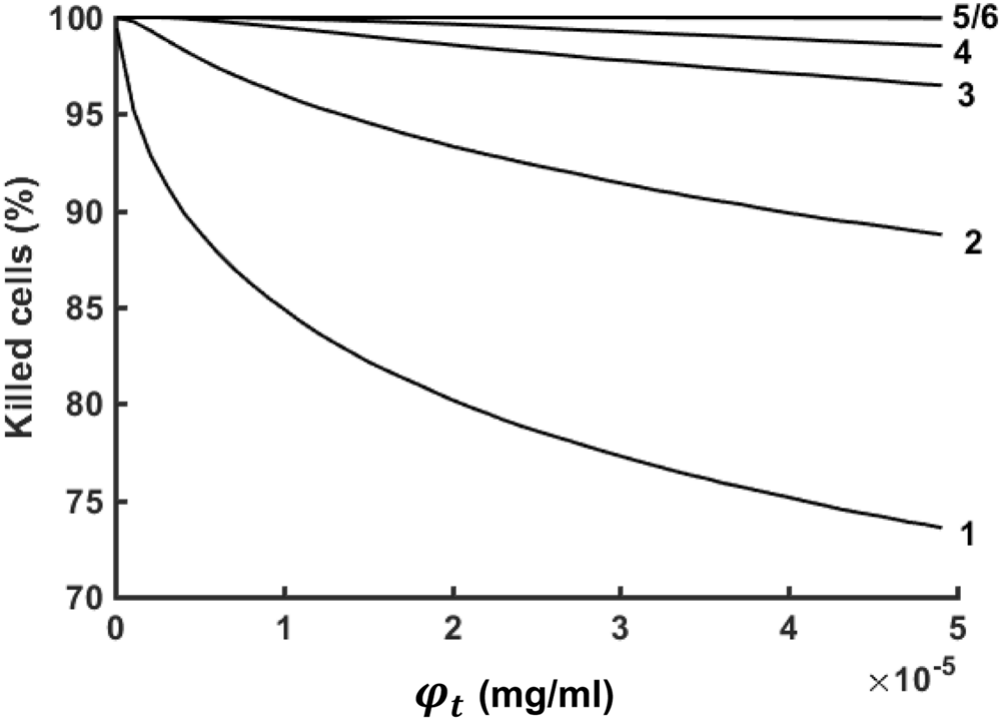
Percentage of killed carcinogenic cells as a function of threshold lethal concentration when a dose of 40 mg cisplatin is equally apportioned between 1 to 6 injections given at optimal locations in the tumor.

Another aspect of the benefits of multiple optimally-placed cisplatin injections is revealed in Fig. 5 which shows the predicted fraction of tumor cells that would be killed as a function of the lethal threshold concentration ${\phi }_{t}$ following a total cisplatin dose of 40 mg of cisplatin, which is the dose currently being used for EBUS-TBNI clinically. A single injection of cisplatin at this dose fails to kill all tumor cells at the lowest value of ${\phi }_{t}={10}^{-7}$ mg.ml^−1^, and by ${\phi }_{t}=5\times {10}^{-5}$ mg.ml^−1^ less than 75% of the tumor has been eradicated. Indeed, complete cell killing is not achieved with a single injection until ${\phi }_{t}$ falls to $6.54\times {10}^{-9}$ mg/mL^−1^. In contrast, 5 injections are completely effective until ${\phi }_{t}=2.73\times {10}^{-5}$.

**Figure 6:**
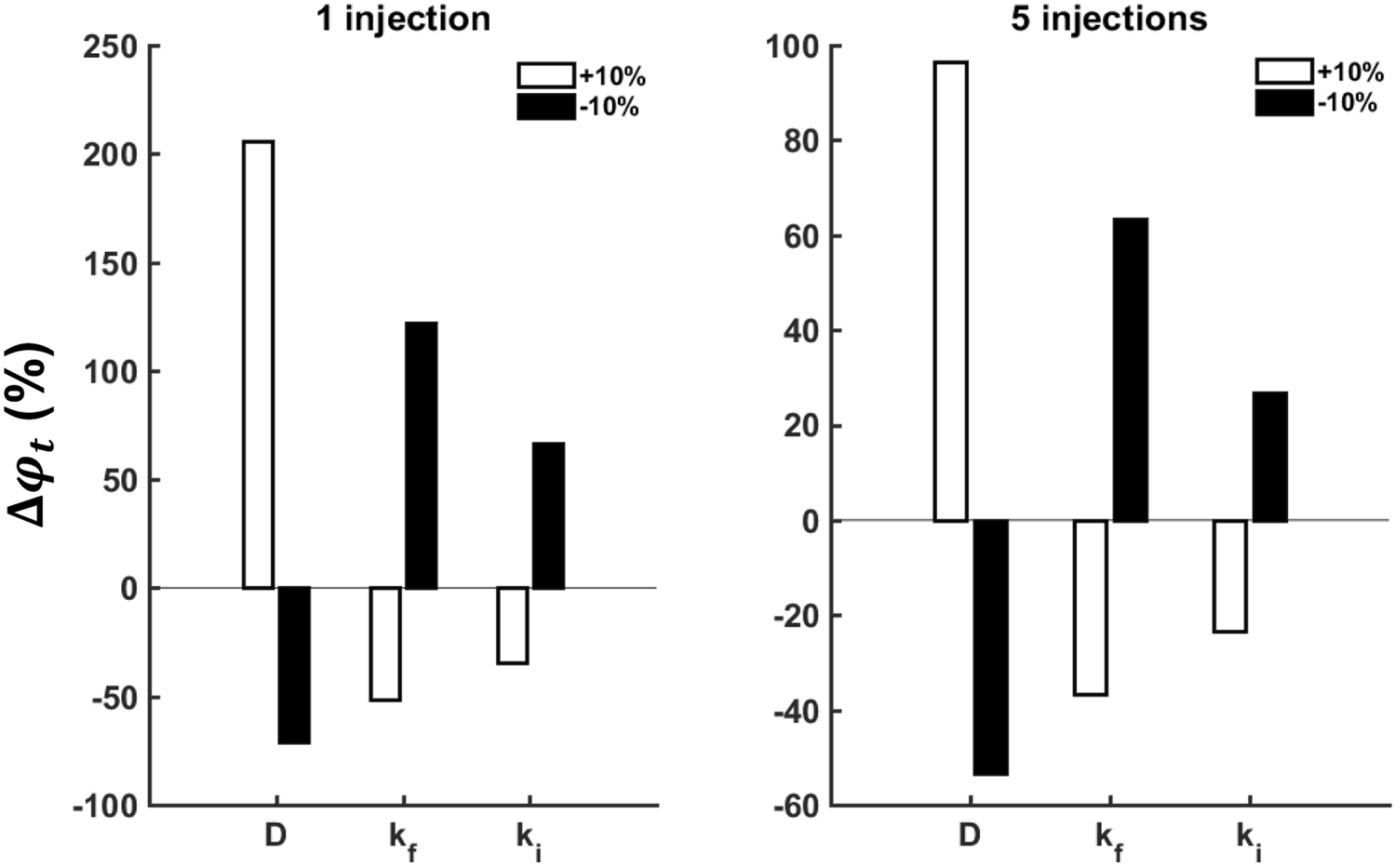
Sensitivity analysis of the model to variations in the three key parameters $D$, ${k}_{i}$ and ${k}_{i}$. For 1 (left) and 5 (right) optimally located injections, each parameter was adjusted in turm by +10% (white bars) and −10% (black bars). With each adjustment the percentage change in the lethal threshold concentration of cisplatin that would result in the death of all tumor cells with a total injected dose of 40 mg was determined.

A sensitivity analysis of the model predictions to the values of the parameters $D,\,{k}_{f}$ and ${k}_{i}$ was performed by adjusting each parameter in turn by ±10% of its best-fit value and then determining how this affected the maximum value of ${\phi }_{t}$ at which complete tumor killing was achieved with a total cisplatin dose of 40 mg. Figure 6 shows these calculations for 1 and 5 injections. The parameter sensitivies are substantially less for 5 injections than for a single injection, again speaking to the relative advantages of the multiple injection strategy. This analysis also shows that increasing the rate of diffusion within the intracellular space raises ${\phi }_{t}$ and thus permits complete killing with a reduced dose of cisplatin. This is not surprising since more rapid spread of cisplatin to sites within the tumor that are distant from the sites of injection will allow concentrations at the distant sites to rise to higher levels before the drug is cleared. Conversely, increasing either ${k}_{f}$ or ${k}_{i}$ causes the value of ${\phi }_{t}$ to decrease, presumably because increasing the loss of drug to sinks near the injection sites reduces the amount left to diffuse to distant parts of the tumor.

## Discussion

EBUS-TBNI of cisplatin has recently emerged as an alternative treatment for peribronchial lung tumors, the motivation being to achieve high intratumoral concentrations while reducing harmful off-target side effects. There is currently no consensus as to how cisplatin should be delivered in to a tumor, nor is it known how injection strategy impacts treatment efficacy. It nevertheless seems reasonable to suppose that efficacy should depend on the number, location and dose of individual injections. Determining the optimal injection strategy for a given tumor is, however, a very non-trivial task given the number of disparate factors that come into play. These factors include tumor volume and shape, the nature of the perfusing vasculature, and features of the tumor tissue including its density and the orientation of fascial planes. While not all of these can be determined in a noninvasive fashion, tumor shape is accurately resolved in a CT scan. We exploited this opportunity in the present study to investigate how cisplatin might distribute itself throughout the tumor from a number of specified injection sites, albeit in a model that approximates reality in numerous ways not the least of which is the assumption that the tumor tissue is biophysically homogeneous and isotropic. Nevertheless, this simple model provides an accurate accounting of cisplatin in the blood as a function of time following EBUS-TBNI of cisplatin into the tumor of a patient with lung cancer (Fig. 2). The model yields a value for the extra-tumoral volume of distribution ($${V}_{f})$$ of 12.2 L, which is similar to volume of the extracellular fluid compartment in a 70 kg adult man; this volume is known to have a value in L that is approximately 20% of body weight in kg^12^. The model also yields a value for $${k}_{f}$$ that is similar to published values of *k*_*i*_^10,11^, which is not unexpected given that these two rate constants reflect rates of diffusion between different compartments of the same tumor tissue. Our model thus appears to capture the overall nature of cisplatin kinetics within the body and consequently has the potential, at the very least, to help quantify systemic exposure to this noxious drug.

The most important finding of our study, however, is the enormous apparent benefit of apportioning a given dose of cisplatin between a number of well-placed injections rather than delivering the entire dose into a single central location, as shown in Fig. 3. Indeed, our model predicts that the dose of cisplatin required to kill a given fraction of tumor cells using 5 injections can be 3 orders of magnitude less than that required for a single injection (Fig. 4). At 6 injections we appear to be approaching the point of diminishing returns, but these results provide compelling evidence that EBUS-TBNI should not be limited to a single injection site in the treatment of lung cancer. This conclusion is further supported by the results shown in Fig. 5 which indicate that increasing the number of injections has a marked effect on the robustness of treatment efficacy in the presence of variations in the local lethal concentration of cisplatin; 5 or more injections are predicted to be almost uniformly efficacious over the range of ${\phi }_{t}$ studied while a single injection is relatively fragile in this respect. Of course, these results are predicated on the cisplatin injections being delivered at the optimal sites predicted by our model. On the other hand, the locations of these optimal sites are distributed roughly uniformly throughout the body of the tumor (Fig. 3). It may therefore be that empirical placement of injections guided simply by the principle of uniform distribution will be close enough to optimal that most of the predicted gains of multiple injections will be realized.

We had to assign values to certain key parameters in the model based on best guesses from the literature, such as a diffusion coefficient reported in normal tissues^9^ and an intracellular rate-constant matched to values reported for neck and gastric tumors^10,11^. There will always remain uncertainty in these values, not to mention the fact they they may exhibit significant spatial variations within a given tumor. The parameter sensitivity analysis presented in Fig. 5 shows that our model predictions can be rather sensitive to errors and/or uncertainties in these parameters. Indeed, 10% variations in the parameter $D$ can affect predictions of tumor killing by as much as 200% (Fig. 5A). We thus cannot expect the predictions of the model to provide precise guidelines as to the total dose of cisplatin to administer to any particular tumor. In fact, it seems reasonable to suggest that a margin of safety of several fold above the predicted minimal dose would be clinically advisable. Nevertheless, an increase of several-fold in the total dose given in 5 well-placed injections is still vastly less than the 3 orders of magnitude increased dose required in a single injection, underscoring the apparent importance of distributing the initial cisplatin load at multiple sites throughout the tumor.

Our study has a number of limitations that reflect the myriad assumptions made in the computational model, many of which have already been alluded to. These limitations will be reduced as we develop the means to characterize the internal structure of lung tumors in greater detail than is possible at present so that the clearly unrealistic assumptions of homogeneity and isotropy can be relaxed. Another potential limitation of our model concerns the way in which we link local cisplatin concentration, ${\phi }_{i}(\overrightarrow{r},t)$, to tumor cell killing, a matter that is still somewhat controversial. For example, Kurihara *et al*.^10^ reported a dependence of cytotoxicity on peak ${\phi }_{i}(\overrightarrow{r},t)$ in human gastric cancer cell lines. Others, however, have suggested that cytotoxicity depends on the area under the concentration-time curve (AUC)^13,14^, on AUC between specified time points^15,16^, and on total exposure time^17^. Several models of tumor cell survival relative to control cells have proposed a dependence on AUC^14^, peak cisplatin concentration^18^, and the time-integration of cisplatin concentration raised to some power^1^. In the present study we chose a rather straightforward cytotoxicity function, namely that a cell dies when its total cisplatin load exceeds a specified lethal threshold. This, however, can easily be modified to some other concentration-lethality function should a better alternative come to light.

We also made certain simplifying assumptions in deriving the model equations, such as the volume of the intracellular space being smaller than that of the extracellular space, and neglecting the return of drug to the tumor from the fluid space. These assumptions were made not only in the interests of arriving at analytic solutions to the model equations that are rapidly solvable, but also because cisplatin binds irreversibly to DNA. There nevertheless remains the possibility that the cytoplasmic cisplatin concentration could rise to the level where it starts to efflux out of the cell before having a chance to bind to the DNA. However, Alborzinia *et al*.^19^ found that cisplatin-treated cells demonstrated stepwise decrements in mitochondrial respiration with increasing concentrations of cisplatin above 5.0 uM, implying that this was below the concentration at which all binding sites are saturated. In our study we injected 133 moles of cisplatin into a tumor having a volume of roughly 40 ml, so even if every molecule of cisplatin was absorbed irreversibly by the tumor cells we would reach a maximum concentration of 3.3 uM. Furthermore, our model indicates that the rate-constants governing flux of cisplatin into the intracellular and vascular spaces from the extracellular space are roughly the same, so the maximum possible intracellular concentration would then be only half of this, or about 1.6 uM, and even this concentration would be achieved only transiently. Thus, it seems likely that the maximum intracellular dose achieved in the model would be well below that needed to saturate all cisplatin binding sites for the majority of the time following injection, making the cisplatin efflux from intracellular to extracelluar spaces correspondingly small. We also assumed the tumor tissue to be heterogeneous and isotropic due to absence of knowledge of these details, but it is likely that neither assumption approximates the truth. These unrealistic assumptions can be relaxed as we develop the means to characterize the internal structure of lung tumors in greater detail than is possible at present. Inclusion of these extra details will require the use of fully numerical methods to solve the model equations. This might provide more accurate predictions of minimal cisplatin dose and optimal injection locations, although it remains to be seen whether these gains in precision will be clinically important.

Another major limitation of our study is the fact that we tested the model predictions against data from a single patient, and thus can consider the model to have been tested in only a very preliminary manner. A convincing validation of the model will obviously require data from a number of study subjects. We are limited in this regard at the present time, because EBUS-TBNI as a treatment modality is still in the very early stages of development, and indeed is only used currently as a salvage therapy for lung cancer patients that have previously failed both radiation and chemotherapy. Consequently, there are very few patients available in whom to make measurements. A key hope of the present study is that by improving the optimization of EBUS-TBNI we will be able to increase the scope of its indications, which will eventually increase the number of patients receiving this therapy and thus make more detailed testing of our model possible. In the meantime, our goal is to introduce the model and to demonstrate, in principle, how it might be useful. The sensitivity analyses we performed (Figs 4–6) go some way toward addressing how the model performs under various situations.

In conclusion, we have developed a mathematical/computational model of cisplatin pharmacodynamics that allows us to predict the distribution and ultimate fate of cisplatin delivered to a lung tumor via EBUS-TBNI. We used the model to predict the minimal efficacious dose of cisplatin and its optimal sites of administration in an accurately reconstructed tumor imaged in a patient with lung cancer. The model gave an accurate fit to measured concentrations of cisplatin in the blood over the 2 hrs following injection. Most importantly, the model predicted that dramatic reductions in the effective dose of cisplatin in this tumor would be possible if the drug was apportioned between 5 appropriately selected sites throughout the tumor rather than being delivered in its entirety at a single central site.

## Data Availability

The experimental data in this manuscript are available from the authors on request.

## References

[1] Adams DJ, Watkins PJ, Knick VC, Tuttle RL, Bair KW (1990). Evaluation of arylmethylaminopropanediols by a novel *in vitro* pharmacodynamic assay: correlation with antitumor activity *in vivo*. Cancer Res.

[2] Olaussen KA (2006). DNA repair by ERCC1 in non–small-cell lung cancer and cisplatin-based adjuvant chemotherapy. New England Journal of Medicine.

[3] Roos WP, Kaina B (2006). DNA damage-induced cell death by apoptosis. Trends Mol Med.

[4] CISplatin Injection. FDA Report Reference ID: 3708516. Report No. 3708516, (https://www.accessdata.fda.gov/drugsatfda_docs/label/2015/018057s083lbl.pdf, 2015).

[5] Khan F, Anker CJ, Garrison G, Kinsey CM (2015). Endobronchial Ultrasound–Guided Transbronchial Needle Injection for Local Control of Recurrent Non–Small Cell Lung Cancer. *Annals of the American Thoracic*. Society.

[6] Mehta HJ (2015). Restoration of patency to central airways occluded by malignant endobronchial tumors using intratumoral injection of cisplatin. Annals of the American Thoracic Society.

[7] el-Khateeb M (1999). Reactions of cisplatin hydrolytes with methionine, cysteine, and plasma ultrafiltrate studied by a combination of HPLC and NMR techniques. J Inorg Biochem.

[8] Chu G (1994). Cellular responses to cisplatin. The roles of DNA-binding proteins and DNA repair. J Biol Chem.

[9] Swabb EA, Wei J, Gullino PM (1974). Diffusion and convection in normal and neoplastic tissues. Cancer research.

[10] Kurihara N (1995). Antitumor activity of cis-diamminedichloroplatinum (II) depends on its time x concentration product against human gastric cancer cell lines *in vitro*. J Surg Oncol.

[11] Troger V, Fischel J, Formento P, Gioanni J, Milano G (1992). Effects of prolonged exposure to cisplatin on cytotoxicity and intracellular drug concentration. European journal of cancer.

[12] Hall, J. E. *Guyton and Hall textbook of medical physiology e-Book*. (Elsevier Health Sciences, 2015).

[13] Nagai N, Ogata H (1997). Quantitative relationship between pharmacokinetics of unchanged cisplatin and nephrotoxicity in rats: importance of area under the concentration-time curve (AUC) as the major toxicodynamic determinant *in vivo*. Cancer Chemother Pharmacol.

[14] Ozawa S, Sugiyama Y, Mitsuhashi Y, Kobayashi T, Inaba M (1988). Cell killing action of cell cycle phase-non-specific antitumor agents is dependent on concentration–time product. Cancer Chemother Pharmacol.

[15] Levasseur LM, Slocum HK, Rustum YM, Greco WR (1998). Modeling of the time-dependency of *in vitro* drug cytotoxicity and resistance. Cancer Res.

[16] Ma J (1994). Pharmacokinetic-dynamic relationship of cisplatin *in vitro*: simulation of an i.v. bolus and 3 h and 20 h infusion. Br J Cancer.

[17] Erlichman C, Vidgen D, Wu A (1987). Antineoplastic drug cytotoxicity in a human bladder cancer cell line: implications for intravesical chemotherapy. Urol Res.

[18] El-Kareh AW, Secomb TW (2003). A mathematical model for cisplatin cellular pharmacodynamics. Neoplasia.

[19] Alborzinia H (2011). Real-time monitoring of cisplatin-induced cell death. PLoS One.

